# The structural alteration of gut microbiota in low-birth-weight mice undergoing accelerated postnatal growth

**DOI:** 10.1038/srep27780

**Published:** 2016-06-09

**Authors:** Jingjing Wang, Huang Tang, Xiaoxin Wang, Xu Zhang, Chenhong Zhang, Menghui Zhang, Yufeng Zhao, Liping Zhao, Jian Shen

**Affiliations:** 1 Ministry of Education Key Laboratory for Systems Biomedicine, Shanghai Centre for Systems Biomedicine, Shanghai Jiao Tong University, Shanghai, PR China; 2 State Key Laboratory of Microbial Metabolism, School of Life Sciences and Biotechnology, Shanghai Jiao Tong University, Shanghai, PR China

## Abstract

The transient disruption of gut microbiota in infancy by antibiotics causes adult adiposity in mice. Accelerated postnatal growth (A) leads to a higher risk of adult metabolic syndrome in low birth-weight (LB) humans than in normal birth-weight (NB) individuals, but the underlying mechanism remains unclear. Here, we set up an experiment using LB + A mice, NB + A mice, and control mice with NB and normal postnatal growth. At 24 weeks of age (adulthood), while NB + A animals had a normal body fat content and glucose tolerance compared with controls, LB + A mice exhibited excessive adiposity and glucose intolerance. In infancy, more fecal bacteria implicated in obesity were increased in LB + A pups than in NB + A pups, including *Desulfovibrionaceae*, *Enterorhabdus*, and *Barnesiella*. One bacterium from the *Lactobacillus* genus, which has been implicated in prevention of adult adiposity, was enhanced only in NB + A pups. Besides, LB + A pups, but not NB + A pups, showed disrupted gut microbiota fermentation activity. After weaning, the fecal microbiota composition of LB + A mice, but not that of NB + A animals, became similar to that of controls by 24 weeks. In infancy, LB + A mice have a more dysbiotic gut microbiome compared to NB + A mice, which might increase their risk of adult metabolic syndrome.

In clinical practice for low birth-weight (LB) human infants, accelerated postnatal growth, also called catch-up growth, is promoted by providing the neonates with surplus nutrition^1^,^2^. Accelerated postnatal growth increases the childhood survival of these LB neonates and lowers their risk of short stature and subnormal intellectual performance in adulthood^3^. However, both epidemiological and animal studies have shown that accelerated postnatal growth is a risk factor for adult obesity and diabetes; furthermore, human individuals with both LB and accelerated postnatal growth are at a higher risk than subjects that are born with a normal birth-weight (NB) and only have accelerated postnatal growth^4^,^5^,^6^,^7^. Studies with rodents show that both LB and accelerated postnatal growth reduce insulin secretion and/or insulin sensitivity^8^,^9^,^10^, increase lipogenesis and adipocyte size in white adipose tissues^9^,^11^, disturb the development of the hypothalamic-pituitary-adrenal (HPA) axis^12^,^13^, and induced epigenetic changes in genes involved in metabolic control^14^,^15^. Despite these efforts, the mechanism underlying the higher risk of adult metabolic diseases of LB individuals with accelerated postnatal growth compared to normal subjects and NB individuals with accelerated postnatal growth needs to be further explored.

Gut microbiota play a pivotal role in obesity and diabetes^16^,^17^,^18^. Recent studies in mice and humans showed that the disruption of gut microbiota composition in infancy produced long-lasting effects on the host metabolism in later life. In mice, the transient disruption of infant gut microbiota from one week before birth to weaning by low-dose antibiotic treatment plays a causative role in excessive adiposity in adulthood, because the transplantation of the antibiotic-disrupted gut microbiota, characterized by decreased levels of *Lactobacillus*, *Allobaculum* and *Rikenellaceae*, to germ-free mice resulted in adiposity in the recipients^19^. In humans, compared with children remaining a normal weight at 7 years of age, children becoming overweight at this age had more *Staphylococcus aureus* and less *Bifidobacterium* spp. in their feces during infancy^20^; besides, the intake of the probiotic *Lactobacillus rhamnosus* GG by mothers in the final 4 weeks of pregnancy and, subsequently, by infants from birth to 6 months of age restrained the excessive weight gain of infants during the first two years of life, and lowered the body mass index (BMI) of overweight children at 10 years of age^21^. Because both LB due to restricted utero nutrition and accelerated postnatal growth due to postnatal overfeeding are early life events, the relevance of gut microbiota with adult obesity and diabetes of LB individuals undergoing accelerated postnatal growth remains to be elucidated.

Studies in humans have compared the composition of the fecal microbiota of preterm LB infants with that of full-term NB infants within months after birth^22^,^23^. LB infants had increased levels of Gammaproteobacteria^24^, *Enterobacteriaceae*, *Enterococcus*, *Staphylococcus*^25^,^26^,^27^, and pathogens such as *Clostridium difficile* and *Klebsiella pneumoniae*^28^,^29^, and reduced *Bifidobacterium*^28^. However, these human studies did not follow up on the metabolic health and gut microbiota composition of LB individuals in later life; thus, the link between infancy gut microbiota and childhood/adult diseases is unclear^22^. Moreover, these human studies are limited by the common treatment of empirical broad-spectrum antibiotics to the preterm/LB infants during the first days after birth, which can disturb gut microbial colonization^23^. In LB rats, Fanca-Berthon *et al*. found that the cecocolonic microbial composition of LB rats differed from that of NB rats from age of day 5 (infancy) to day 100 (adulthood)^30^. However, they screened limited number of bacterial groups/species with quantitative real-time PCR, and provided incomplete profiles of gut microbiota composition. Therefore, the alterations in gut microbiota of LB individuals with accelerated postnatal growth need to be further explored.

In the present study, we generated NB and LB mouse neonates by subjecting the pregnant dams to *ad libitum* food access and 50% food intake restriction in late gestation^10^, respectively, and over-fed the neonates and thus induced accelerated postnatal growth (A) by reducing the litter size during lactation^31^ (Supplementary Figure S1). We compared the metabolism and gut microbiota structure of LB + A mice, NB + A mice, and control mice with NB and normal postnatal growth during infancy and adulthood. The results showed LB + A mice had more dysbiotic gut microbiome compared to NB + A mice at infancy, and this was associated with the increased risk of adult metabolic syndrome of LB + A mice compared to NB + A mice. This study provides a novel insight into the possible role of infant gut microbiota in adult metabolic syndrome of LB + A individuals.

## Results

### The effects of postnatal overfeeding on the growth of LB and NB mice

**Figure 1:**
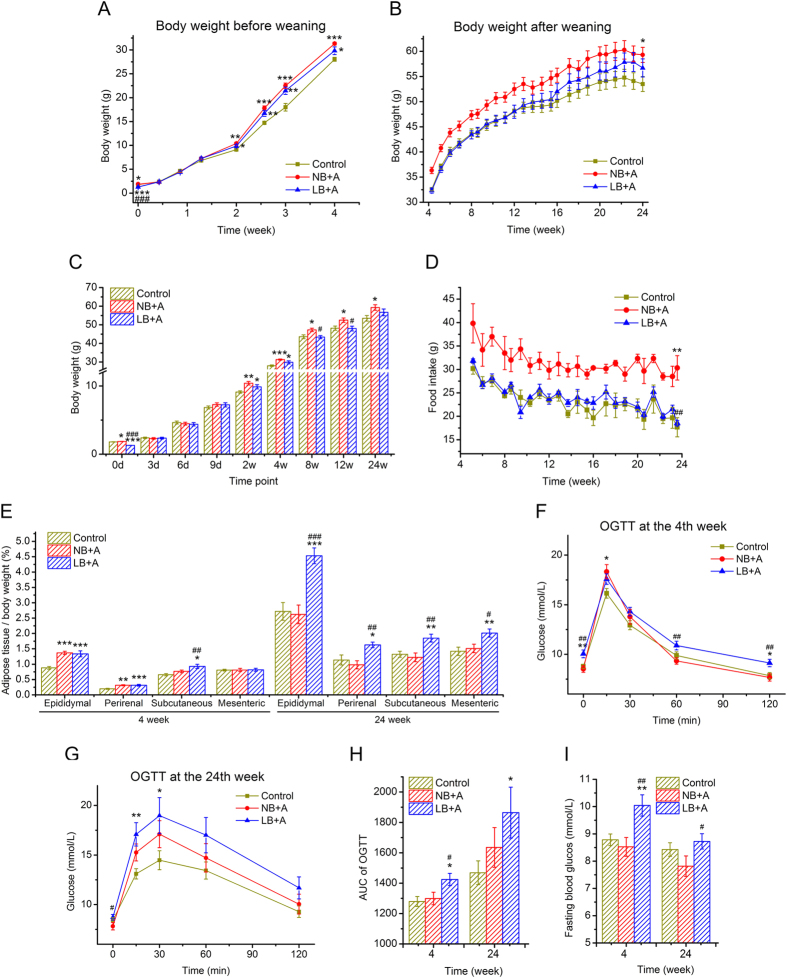
The effects of suckling overfeeding-induced accelerated postnatal growth on the growth and metabolism of low-birth-weight (LB) and normal-birth-weight (NB) mice. (**A**) The body weight curve from birth to age 4 weeks (weaning). (**B**) The body weight curve from age 4.5 weeks to 24 weeks. (**C**) Body weight at representative time points. d, days; w, weeks. (**D**) Food intake after weaning at 4 weeks. (**E**) Adipose pads as a percentage of body weight at 4 and 24 weeks of age. (**F**) Curve of the Oral Glucose Tolerance Test (OGTT) at 4 weeks of age. (**G**) Curve of OGTT at 24 weeks. (**H**) Areas under the curve (AUC) of OGTT at 4 and 24 weeks. (**I**) Fasting blood glucose at 4 and 24 weeks. Data are shown as the means ± SEM. Differences were assessed by ANOVA. *P < 0.05, **P < 0.01, ***P < 0.001 *vs.* control group; ^#^P < 0.05, ^##^P < 0.01, ^###^P < 0.001 *vs.* NB + A group. n = 12 per group.

Because the dams of LB + A pups were subjected to 50% restriction of food intake in late pregnancy, the neonatal LB + A pups weighed on average 0.5 to 0.6 g less than control and NB + A pups at birth (LB + A, 1.28 ± 0.02 g; Control, 1.80 ± 0.02 g; NB + A pups 1.86 ± 0.02 g; p < 0.001 LB + A *vs*. Control; p < 0.001 LB + A *vs*. NB + A) (Fig. 1A,C, Supplementary Table S1). The 50% restriction of food intake in late pregnancy did not reduce the pup number per litter (Supplementary Table S2).

During lactation (from birth to age 28 days), the overfeeding induced by litter size reduction led to accelerated postnatal growth of LB and NB pups. NB + A pups weighed similarly to control pups from birth to 9 days of age (p > 0.05), and then weighed significantly more (p < 0.01) than did the control mice from 2 weeks of age to weaning at 4 weeks (Fig. 1A,C, Supplementary Table S1). The body weight of the LB + A pups reached the weight of the control and NB + A pups by 3 days of age, then remained similar to that of control and NB + A pups from 6 days to 9 days of age; from 2 to 4 weeks of age, LB + A pups weighed significantly higher than control mice, but tended to weigh 0.5 g, 1 g, and 1.5 g less than the NB + A animals at age 2 weeks (p = 0.149), 3 weeks (p = 0.312) and 4 weeks (p = 0.073), respectively (Fig. 1A,C, Supplementary Table S1).

After weaning (from 29 days to 24 weeks), all of the animals were fed a normal chow diet *ad libitum*. While NB + A mice showed a persistent 30% increase in food intake compared with control and LB + A mice, LB + A animals ingested an equivalent amount of chow as control mice (Fig. 1D). NB + A mice steadily weighed more than control mice by approximately 10% after weaning (Fig. 1B,C, Supplementary Table S1). From the age of 30 days to 12 weeks, the body weight of LB + A animals was equal to that of controls and remained significantly lower than that of NB + A mice. The body weight of LB + A mice started to increase from 12 weeks and was between that of the control and NB + A mice at 24 weeks, but this value was not significantly different from that of either the control or NB + A groups (Fig. 1B,C, Supplementary Table S1).

### LB + A mice had excessive adiposity at both age 4 and 24 weeks, whereas NB + A mice did only at age 4 weeks

At 4 weeks of age (before weaning), both LB + A and NB + A mice had significantly higher percentages of epididymal and perirenal fat than control mice, and the percentages were not different between LB + A and NB + A mice (Fig. 1E). The percentage of subcutaneous inguinal fat of LB + A pups was higher than that of the control and NB + A mice (Fig. 1E). The absolute masses of epididymal, perirenal and subcutaneous inguinal fat pads of LB + A and NB + A pups were significantly higher than those of control pups, but there was no difference between LB + A and NB + A pups (Supplementary Table S3).

At 24 weeks of age, the proportions or absolute mass of epididymal, perirenal, mesenteric and subcutaneous inguinal adipose pads of NB + A mice were not different from those of control mice (Fig. 1E, Supplementary Table S3). In contrast, LB + A mice had disproportionally higher amounts of epididymal, perirenal, mesenteric and subcutaneous inguinal adipose pads than did the other two animal groups (Fig. 1E, Supplementary Table S3).

### LB + A mice were glucose intolerant at 4 and 24 weeks of age, whereas NB + A mice were not

At 4 weeks, while LB + A animals showed significantly impaired glucose clearance in the OGTT and increased fasting glucose level compared with control and NB + A animals, NB + A animals had a normal capacity to clear up glucose bolus in OGTT and a normal fasting glucose level, similar to the control group (Fig. 1F,H,I).

At 24 weeks, the area under the OGTT curve of LB + A mice was significantly higher than that of control mice, but the values for NB + A mice were not different from those of the control animals (Fig. 1G,H). The area under the OGTT curve of NB + A mice tended to be lower than that of the LB + A counterparts, but the difference did not reach significance (p = 0.23, Fig. 1G,H). Moreover, LB + A mice had higher fasting glucose levels than did the NB + A animals at 24 weeks (Fig. 1I).

### Comparison of overall structural of gut microbiota among LB + A, NB + A and control mice

Bar-coded 454 pyrosequencing of bacterial 16S rRNA gene V3 region was performed for 106 fecal samples from three groups of mice at 4, 12 and 24 weeks of age. After denoising, a total of 432,428 usable high-quality raw reads were obtained, with an average of 4079 ± 992 reads per sample (Supplementary Figure S2). The sequences were binned into 987 OTUs at the 98% similarity level. After chimeras and singleton filtering, there were 666 OTUs. At this sequencing depth, rarefaction curves did not plateau (Supplementary Figure S3A–C), but the Shannon diversity index for each sample was stable (Supplementary Figure S3D–F), which suggests that most of the diversity had already been covered despite the appearance of rare new phylotypes upon further sequencing. At the ages of 4, 12 and 24 weeks, Shannon diversity indices were not significantly different among the control, LB + A and NB + A mice (Supplementary Figure S3G).

**Figure 2:**
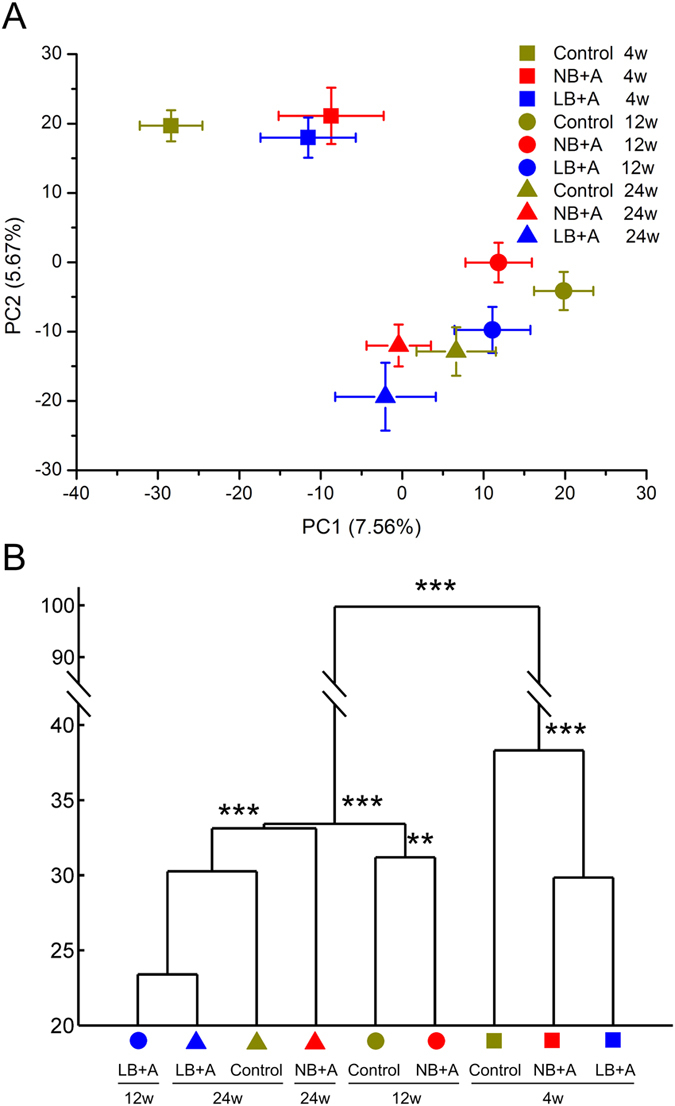
The overall structure of fecal microbiota of LB + A, NB + A and control mice at 4, 12 and 24 weeks of age. (**A**) PCA scores plot based on OTU abundance. Each point represents the mean principal coordinate (PC) score of all mice in an animal group at one time point, and the error bar represents the s.e.m. (**B**) The clustering of gut microbiota of different animal groups calculated with multivariate analysis of variance (MANOVA) test, and *P < 0.05, **P < 0.01, ***P < 0.001. wk, weeks. n = 11 for control group at age 4 and 24 weeks; n = 12 for control group at age 12 weeks and other two group at three time points.

Based on the OTU abundance matrix, a Principal Components Analysis (PCA) that includes all samples of three animal groups at 4, 12 and 24 weeks of age was performed. The scores plot of PC1 and PC2 (Fig. 2A) revealed that the major separation of gut microbiota composition was between infancy (age 4 weeks, before weaning) and adulthood (age 12 and 24 weeks), regardless of the group origin of samples, indicating that ageing and dietary changes (breast feeding in infancy, normal chow diet in adulthood) were the major factors influencing the overall structure of gut microbiota.

To cluster the gut microbiota of the animals based on structural similarity, and to test the difference in fecal microbiota composition between animals groups, Multivariate analysis of variance (MANOVA) was performed with multiple PCs explaining over 70% variance of the data (Fig. 2B). Consistent with the scores plot of PC1 and PC2, MANOVA clustering analysis also showed the gut microbiota of 4-week-old mice was significantly different from that of 12- and 24-week-old animals. At 4 weeks, the structures of gut microbiota of LB + A and NB + A pups were significantly different from that of controls. For adult control and NB + A mice, the gut microbiota at 12 weeks clustered separately from the microbiota at 24 weeks, and NB + A mice was significantly different from control mice at both time points. Being significantly different from the microbiota of 12-week-old NB + A and control mice, the gut microbiota of 12-week-old LB + A mice clustered together with 24-week-old LB + A and control mice, and there was no significant different among 12-week-old LB + A, and 24-week-old LB + A and control mice. This indicates the gut microbiota composition of LB + A mice developed in an accelerated manner after weaning, and finally normalized with that of control mice by age 24 week.

### Pair-wise comparison of gut microbiota composition at the phylotype level among LB + A, NB + A and control mice

**Figure 3:**
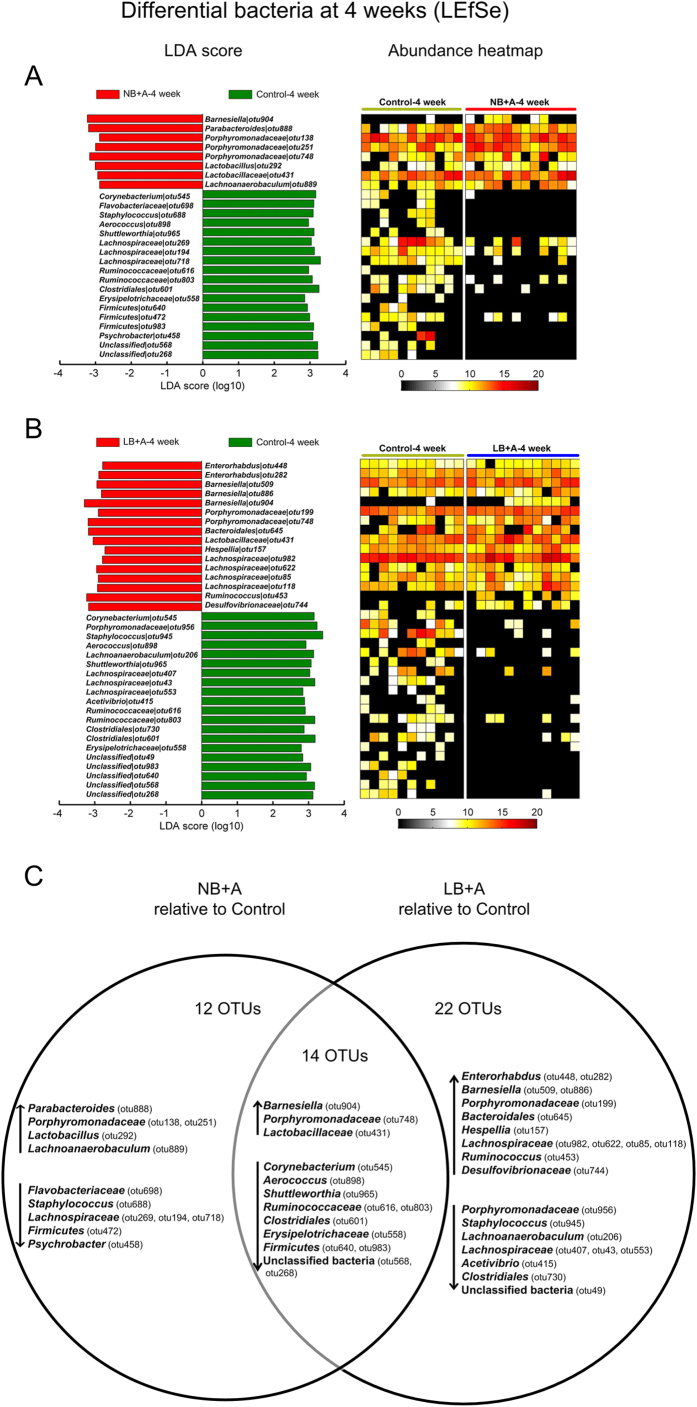
Bacterial phylotypes of which abundances were changed in LB + A and NB + A mice compared to control mice at 4 weeks identified by LEfSe. (**A**) NB + A versus control mice. (**B**) LB + A versus control mice. The left histogram shows the LDA scores computed for OTUs differentially abundant between two animal groups. The right heat map shows the relative abundance (log 2 transformed) of altered OTUs. (**C**) Venn diagrams of OTUs changed in LB + A and NB + A pups relative to control animals at age 4 weeks. The OTU taxonomy is listed. ↑ and ↓ indicate increased and decreased taxa, respectively, relative to control mice.

**Figure 4:**
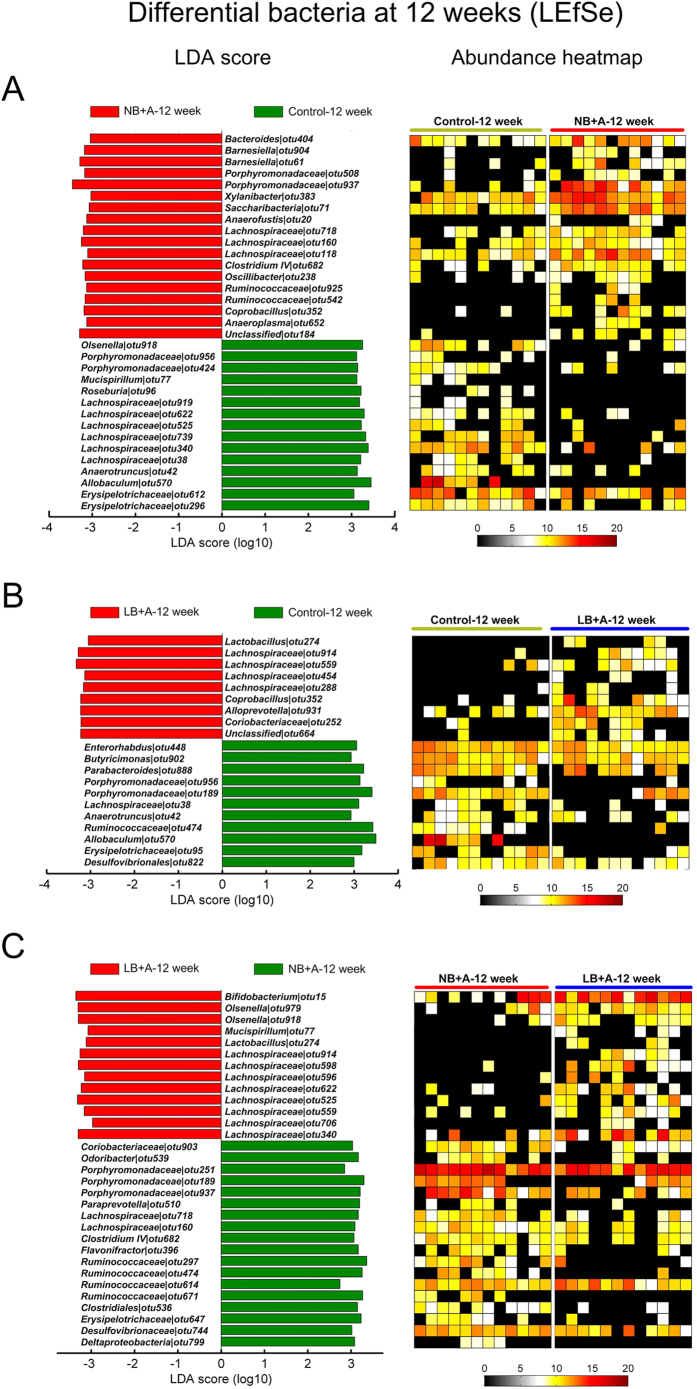
Bacterial phylotypes of which abundances were different between two animal groups among 12-week-old LB + A, NB + A and control mice identified by LEfSe. (**A**) NB + A versus control mice. (**B**) LB + A versus control mice. (**C**) LB + A versus NB + A mice. The left histogram shows the LDA scores computed for OTUs differentially abundant between two animal groups. The right heat map shows the relative abundance (log 2 transformed) of altered OTUs.

**Figure 5:**
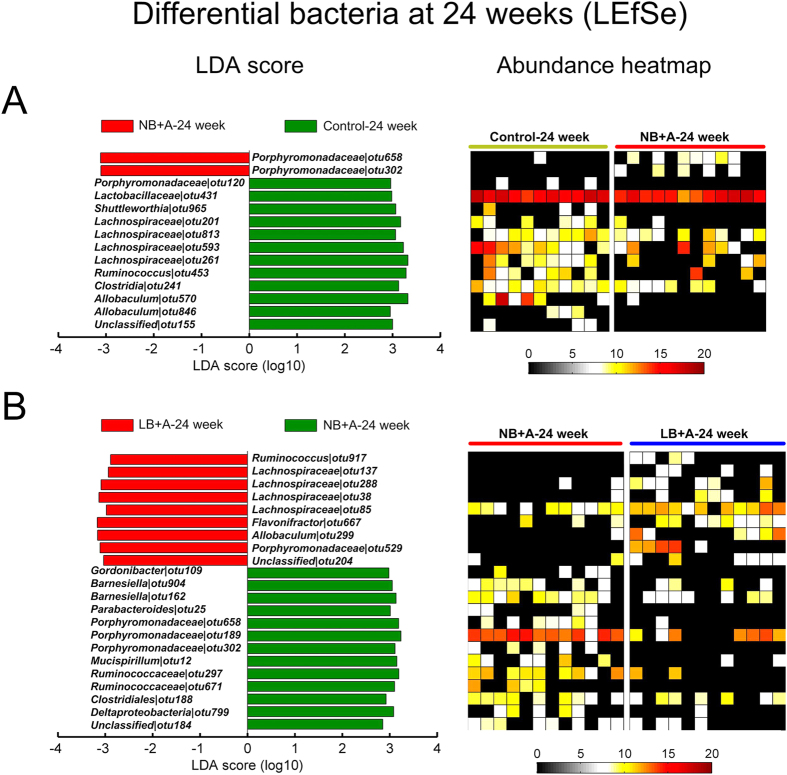
Bacterial phylotypes of which abundances were changed in NB + A mice compared to control mice, and in LB + A mice compared to NB + A mice at age 24 weeks identified by LEfSe. (**A**) NB + A versus control mice. (**B**) LB + A versus NB + A mice. The left histogram shows the LDA scores computed for OTUs differentially abundant between two animal groups. The right heat map shows the relative abundance (log 2 transformed) of altered OTUs.

At the individual time points of 4, 12 and 24 weeks of age, for any two of the three animal groups between which the overall structure of fecal microbiota differed according to the MANOVA tests, a linear discriminant analysis (LDA) effect size (LEfSe) was employed to identify specific phylotypes for which the abundance was different between the groups of mice (Figs 3, 4, 5).

At 4 weeks (before weaning), compared with the control group, LB + A mice had 16 increased and 20 decreased OTUs (a total of 36 OTUs changed), and NB + A mice had 8 increased and 18 decreased OTUs (a total of 26 OTUs changed) (Fig. 3A,B). Only 14 OTUs were commonly altered in both groups, for instance, 1 OTU from *Barnesiella* and 1 OTU from *Corynebacterium* were increased and decreased, respectively, in both LB + A and NB + A pups (Fig. 3C). 22 and 12 OTUs were changed only in LB + A and only in NB + A mice, respectively. Among these bacterial phylotypes, OTUs belonging to *Enterorhabdus*, *Desulfovibrionaceae* and *Barnesiella* were enriched only in LB + A pups, whereas one bacterium from *Lactobacillus* was increased in the NB + A pups but not in LB + A mice (Fig. 3C). These results indicate that the gut microbiota composition of LB + A and NB + A animals was differentially altered in infancy.

At 12 weeks, LB + A and NB + A mice had, respectively, 20 and 33 altered OTUs compared with the control group (Fig. 4A,B). For example, LB + A animals had one *Lactobacillus* bacterium enhanced, whereas NB + A mice uniquely had increased representatives from *Barnesiella* and *Oscillibacter* (Fig. 4A,B). The abundances of a total of 31 OTUs were changed in LB + A mice compared with NB + A mice, and notably, representative from *Bifidobacterium* and *Lactobacillus* were higher in LB + A mice than in NB + A mice (Fig. 4C).

At 24 weeks of age, LEfSe was performed between LB + A and NB + A mice, and between the NB + A and control mice (Fig. 5). Compared with mice belonging to the NB + A group, LB + A mice had 22 OTUs altered, with 9 (including bacteria from *Allobaculum*) increased, and 13 (including *Barnesiella*) decreased (Fig. 5B). Compared with the control animals, NB + A had 2 OTUs (e.g., phylotypes belonging to *Porphyromonadaceae*) increased and 12 OTUs (e.g., phylotypes belonging to *Allobaculum*) decreased (Fig. 5A).

### LB + A mice, but not NB + A mice, showed disrupted gut microbiota fermentation activity compared with control animals in infancy

**Figure 6:**
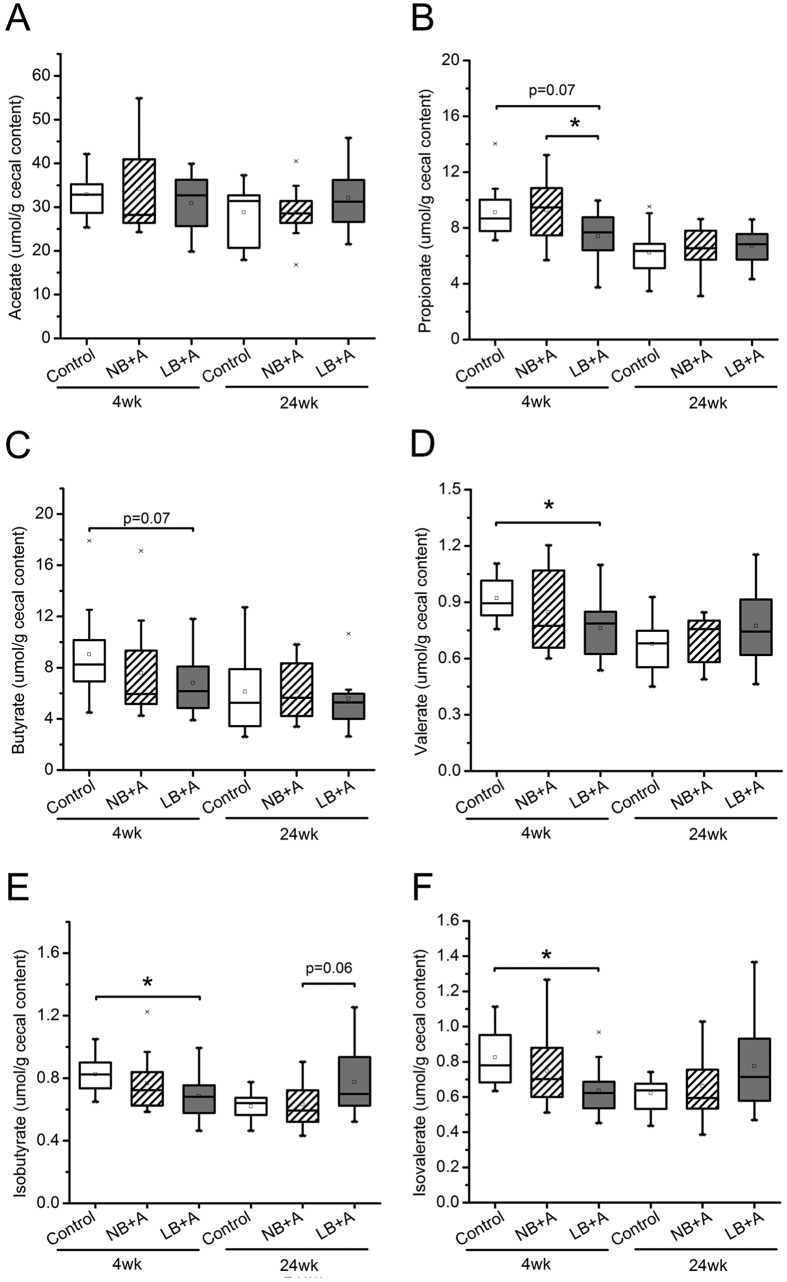
LB + A mice had disrupted fermentation activity of cecal microbiota at age 4 weeks. (**A–F**) Concentrations of acetate (**A**), propionate (**B**), butyrate (**C**), valerate (**D**), isobutyrate (**E**), and isovalerate (**F**) in the cecal contents of LB + A, NB + A and control mice at 4 and 24 weeks of age. In the box plot, the bottom and top are the 25th and 75th percentile, respectively. A line within the box marks the median, and a circle in the box shows the mean. Whiskers above and below the box indicate the 1.5 interquartile range of the lower and upper quartile, and samples beyond are regarded as outliers. Differences were assessed by the Mann-Whitney test. *P < 0.05. wk, weeks.

We measured the cecal concentrations of the short chain fatty acids (acetate, propionate, butyrate and valerate) and the branch chain fatty acids (isobutyrate and isovalerate) in all mice, which reflect the fermentation activity of gut microbiota on carbohydrates and proteins, respectively (Fig. 6). In NB + A mice, the concentrations of these of bacterial fermentation end products were not different from those in control mice at either 4 or 24 weeks. In 4-week-old LB + A mice, the cecal concentrations of propionate and butyrate tended to be lower (p = 0.07), and the cecal levels of valerate, isobutyrate, and isovalerate were significantly lower than those of the control mice pups (p < 0.05). The concentration of propionate was significantly lower in 4-week-old LB + A mice than age-matched NB + A mice. At 24 weeks, the cecal concentrations of these short chain fatty acids and branch chain fatty acids in LB + A mice no longer differed from those in control mice.

## Discussion

In the present study, LB + A mice were more predisposed to adult adiposity and glucose intolerance than were NB + A mice, and this predisposition is associated with the more dysbiotic gut microbiota in infant LB + A mice compared with their NB + A counterparts, which is reflected by three aspects. (1) More bacteria associated with metabolic syndrome occurrence were enriched in LB + A pups than in NB + A pups at 4 weeks of age. Compared with control animals, *Desulfovibrionaceae*, *Enterorhabdus*, and *Barnesiella*, which are implicated in the development of obesity, insulin resistance, and hepatic steatosis in rodents and/or humans^32^,^33^,^34^,^35^, were increased in the LB + A pups but not in the NB + A pups. (2) One *Lactobacillus* bacterium was increased only in NB + A pups but not in LB + A pups. *Lactobacillus* species in the infant mouse gut are implicated in prevention of adult adiposity, because the reduction of these bacteria due to antibiotic treatment in infancy is associated with adult adiposity^19^. (3) Compared to control mouse pups, LB + A but not NB + A pups showed significant decrease in cecal concentration of valerate, iso-valerate and iso-butyrate, and the tendency of decreased propionate and butyrate concentration, which might influence the gut development. In rodents, propionate, butyrate and valerate, but not acetate, stimulate colonic contractions^36^ and regulate proliferation of intestinal epithelial cells^37^. The reduction of cecal iso-valerate and iso-butyrate concentrations in LB + A pups indicates the decreased protein fermentation by the gut microbiota, and thus indicates the decreased generation of other compounds in protein fermentation such as ammonia and indoles that are usually considered toxic to host^38^, however, the effects on gut health of reduced protein fermentation by gut microbiota to the level lower than that of normal control animals remain to be illustrated. Indeed, at physiological concentration in the colon lumen, ammonia stimulates epithelial cell proliferation in rats^39^, and indole strengthens the barrier function and attenuates inflammation of colonic epithelial cells^40^. After weaning, although the fecal microbiota of LB + A mice normalized in composition and fermentation activity to that of control mice by 24 weeks, and LB + A mice harbored higher levels of gut *Lactobacillus*, *Bifidobacterium* and *Allobaculum* species, which are shown to protect against obesity and diabetes^41^,^42^,^43^, than did NB + A mice, this did not normalize/reduce their adult adiposity or diabetes at 24 weeks. These results indicate that gut microbiota disruption in infancy might play an important role in adult adiposity and diabetes of LB + A individuals.

There are several possible mechanisms by which the infant gut microbiome may influence adult metabolism. The infant gut microbiota dysbiosis may disrupt the normal development of the host immune system^44^ and gut barrier function^45^, and may lead to persistent changes in the epigenetic modification of the host genes^46^ during the sensitive postnatal developmental stage. Immune dysfunction and impaired gut barrier have been shown to play a pivotal role in obesity and diabetes^17^,^47^, and early life epigenetic changes have been strongly implicated in later life obesity^14^. In addition, as the founder, the infant gut bacterial composition influences the successional microbiota in later life^22^,^48^, and the gut microbiota changes in LB + A mice relative to control animals after weaning may continuously affect the host metabolism. Indeed, in the present study, the gut microbiota composition of LB + A mice still differed from that of the controls at 12 weeks, though the two groups’ food intakes were equal; Fanca-Berthon *et al*. showed the LB mice due to the protein shortage in the fetal stage had different gut microbiota compositions than did control animals from day 5 to day 100 of life^30^.

In light of our results, it would be interesting to test whether gut microbiota-targeted approaches, especially administrated at infancy, can alleviate adult metabolic syndrome of LB + A individuals. Castaneda-Gutierrez *et al*. administered a mixture of prebiotic bovine milk oligosaccharides, a probiotic *Lactobacillus rhamnosus* strain and long-chain polyunsaturated fatty acids to LB rats from 7 to 58 days of life, and significantly reduced the fat accumulation at 58 days of age^49^. Prebiotics, probiotics and long-chain polyunsaturated fatty acids can modulate the composition and/or metabolism of gut bacteria^42^,^50^,^51^. However, because long-chain polyunsaturated fatty acids can be mostly absorbed and metabolized by the host^52^, it is difficult to attribute the beneficial effects of the mixture to the gut microbiota changes. Therefore, approaches that specifically target the gut microbiota should be tested individually for their capacity to improve adult metabolic syndrome of LB + A subjects, and it would be interesting to compare the effects of these approaches when they are administered in infancy and in adulthood.

Despite of the persistent hyperphagia and altered gut microbiota composition compared with control animals after weaning, NB + A mice had a normal body fat percentage and glucose tolerance at 24 weeks of age, and this is associated with the enhancement of gut *Lactobacillus*, implicated in prevention of adult adiposity^19^, in infant NB + A mice. However, these results do not implicate that NB + A mice are protected from metabolic diseases life long. At both age 12 and 24 weeks, fecal *Allobaculum* bactertia, which are shown to be potentially beneficial for host metabolic health^42^,^53^, were consistently lower in NB + A mice than in control animals. Besides, increase in serum concentration of lipopolysaccharide binding protein (LBP) has been shown to be a risk factor for obesity, type 2 diabetes and related disorders^54^,^55^; here, likely because of the persistent higher food intake after weaning^56^, 24-week-old NB + A mice tended to have higher serum LBP levels than the control animals (Supplementary Figure S4), indicating the potential of NB + A mice to develop metabolic diseases at an older age. Previously, researchers overfed NB ICR mouse pups by reducing the litter size from 10 to 3 pups during lactation, and these mice exhibited glucose intolerance as early as 4 months of life^57^.

Consistent with previous reports, our results showed that postnatal overfeeding resulted in persisting hyperphagia after weaning in NB mice^12^,^58^ but not in LB mice^31^. Postnatal overfeeding has been shown to induce a persisting malformation of the hypothalamic neurons that are involved in appetite regulation^59^ and to up-regulate glucocorticoid metabolism^12^. It is possible that postnatally overfed NB and LB mice had different hypothalamic nuclei development or glucocorticoid metabolism.

The limitation of our study is that we only controlled the number of pups per litter (4 pups/litter) but did not strictly control the milk intake during lactation in the NB + A and LB + A animal groups. Our results showed the higher risk of adult metabolic syndrome of LB + A mice compared to NB + A mice, but this is not likely due to the possibility that LB + A pups consumed more milk than NB + A pups during lactation, because our data indicate the LB + A pups probably did not require much more milk than NB + A pups. First, although LB + A pups weighed on average 0.6 g less than NB + A pups at birth, and thus require more energy for catch-up growth to reach the body weight of NB + A pups in the first 3 days of life, afterwards, the body weight of the LB + A pups remained similar to that of NB + A pups from 6 days to 9 days of age, and then tended to weigh 0.5 g, 1 g, 1.5 g less than the NB + A animals at age 2 weeks (p = 0.149), 3 weeks (p = 0.312) and 4 weeks (p = 0.073), respectively. Therefore, the total energy requirement for growth from birth to age 4 weeks may not significantly different between LB + A and NB + A pups. Second, we found while LB + A mice had normal food intake as control mice *right after weaning*, NB + A mice consumed significantly more chow than age-matched LB + A and control mice *right after weaning*; the food intake/appetite usually do not change abruptly, indeed, other investigators have found that the milk intake of NB + A pups has become higher than that of control pups at age 4 days^60^ and become twice of that of control mice at age 14 days^61^; these results in combination suggest our NB + A pups, which showed hyperphagia, were not likely to consume less milk during lactation than the LB + A mice which had normal appetite. Accurate control of the milk intake of rodent pups via artificial feeding with formula is now feasible only in rats but not mice, because it is relatively easy to introduce intragastric cannula in neonatal rats with larger body size and to raise the rat pups in isolation^62^,^63^. In the future, it will be interesting to perform rat experiments in which milk intake was strictly controlled during lactation by artificial feeding, and compare the effects of postnatal overfeeding on LB and NB rats.

In conclusion, we demonstrated the association of dysbiosis of gut microbiota composition in infancy with adult adiposity and diabetes in LB + A mice. These results indicate that gut microbiota disruption during infancy may play a role in the adult metabolic diseases of LB + A individuals and thus potentiate gut microbiota-targeted approaches to alleviate obesity and diabetes induced by LB + A.

## Methods

### Animal experiment and sample collection

All the animal experiments were carried out in accordance with the Guidelines for Care and Use of Laboratory Animals of Shanghai Laboratory Animal Center (SLAC), Chinese Academy of Sciences, Shanghai, China. The experimental protocols were approved by the Bioethics Committee of School of Life Sciences and Biotechnology, Shanghai Jiao Tong University (No. 2011–008). Mice were kept in the animal facilities of Shanghai Laboratory Animal Center (SLAC) with a 12-hour light-dark cycle and a temperature of 22 ± 3 °C.

The design of the animal trial was shown in Supplementary Figure S1. Virgin, 6- to 8-week-old female ICR mice, weighing between 29–35 g, were mated with male ICR mice overnight. The pregnancy was validated and dated by visualizing the vaginal plug. The pregnant dams were caged individually, and randomly assigned to one of three dam groups. Four Control and 6 NB dams were fed with normal chow (containing 5.2% (w/w) fat, 3.2–3.4 kcal/g, from SLAC Inc., Shanghai, China) *ad libitum* through the whole pregnancy (mean food intake: 9.8 ± 0.3 g/day), and they were supposed to deliver normal birth-weight (NB) pups; 12 LB dams accessed the normal chow freely until pregnancy day 11, and then were given 50% of the chow consumed by Control and NB dams from pregnancy day 12 to delivery (mean food intake: 5.2 ± 0.1 g/day), and LB dams were supposed to deliver low birth-weight (LB) pups. At delivery, the birth weight of each pup was measured. Dams were provided with normal chow *ad libitum* during lactation and nursed their own pups. To create the postnatal overfeeding and induce accelerated postnatal growth (A) in NB and LB pups, the litter size of NB and LB dams was adjusted to 4 pups/litter as opposed to 8 pups/litter for the Control dams. Finally, three offspring groups were generated and designated as LB + A, NB + A, and control (normal birth weight and normal postnatal growth). From birth to 4 weeks of age, the body weight was recorded for each pup once every three days.

At 4 weeks of age, for each offspring group, 12 male pups were randomly selected from at least 4 litters and subjected to body weight measurement and an oral glucose tolerance test (OGTT). Fresh fecal samples were collected and immediately stored at −80 °C until analysis. After 5 h of fasting, all blood was collected from the orbital plexus, and serum was isolated by centrifugation at 3000 rpm at 4 °C for 15 min and stored at −80 °C. The pups were sacrificed by cervical dislocation. Cecal contents were collected and snap frozen in liquid nitrogen and then stored at −80 °C until analysis. Epididymal, perirenal, subcutaneous, and mesenteric adipose tissues were excised and weighed by one person blinded to the grouping of animals.

At 4 weeks of age, another 12 male pups per offspring group were randomly selected from at least 4 litters and were weaned and maintained on the chow *ad libitum* until 24 weeks of age. The body weight and food intake were measured once per week. At 12 and 24 weeks of age, fresh stool samples were collected from each mouse, and an oral glucose tolerance test (OGTT) was performed. At 24 weeks of age, these mice were sacrificed, and the fasting blood, cecal content and adipose pads were sampled as described above.

### Oral glucose tolerance test (OGTT)

After 5 h of food deprivation, glucose was administered orally to the mice at a dose of 2.0 g/kg body weight. Blood samples were taken from the tail before and 15, 30, 60, and 120 min after glucose administration, and the blood glucose levels were measured using a blood glucose meter (Accu-Check; Roche Diagnostics, Mannheim, Germany). The blood glucose level before glucose administration represented the fasting glucose level.

### 454 pyrosequencing of fecal bacterial 16S rRNA gene V3 region

Fecal genomic DNA extraction, amplification of the V3 region, and pyrosequencing of PCR amplicons were performed as described previously^32^. Genomic DNA was extracted from fecal samples collected from three groups of mice at age 4, 12 and 24 weeks using bead beating and an InviMag^®^ DNA kit (Invitek, Berlin, Germany). The V3 region of the 16S rRNA gene was amplified from each sample with the extracted fecal DNA as the template in PCR with 20 cycles. The bacterial universal primer pair for the V3 region consisted of the forward primer 5′-NNNNNNNNCCTACGGGAGGCAGCAG-3′ and the reverse primer 5′-NNNNNNNNATTACCGCGGCTGCT-3′. NNNNNNNN was the sample-unique DNA bar code of eight nucleotide sequences. The PCR amplicons from each sample were quantified with PicoGreen fluorescent dye (Thermo Fisher Scientific, Sunnyvale, USA) by using SpectraMax M5 microplate reader (Molecular Devices, San Francisco, USA), and mixed in equal ratios. The 454 sequencing adapters were ligated to the mixed PCR amplicons by blunt-end ligation. Pyrosequencing was performed on a Genome Sequencer FLX platform (Roche Diagnostics, Mannheim, Germany).

### Bioinformatics and statistical analysis of 454 pyrosequencing data

All raw reads were sorted into different samples according to the sample-unique 8-base barcodes. High-quality sequences were selected using the following criteria: (1) primers at both ends should exist, forward primers at the sequencing end must be complete, and only one pair of primers existed in one sequence; (2) forward barcodes must be complete, and exactly matched to the assigned ones; (3) sequence length of variable region ranged from 100 to 200 bp, and (4) sequences in the variable region contained no more than 2N bases. Read sorting and quality filtering were preformed with in-house scripts. Denoise was performed using denoise_wrapper.py in QIIME. Sequence alignment, operational taxonomic units (OTU) clustering, phylogenetic tree construction, alpha-diversity analysis, and beta-diversity analysis were performed using QIIME (v1.8.0)^64^. All high-quality sequences were aligned against Greengenes core dataset with by PyNAST multialigner, and sequences with aligned length ≥100 bases and percent identity ≥75% with the reference sequences were used for further analysis. OTUs were clustered *de novo* with UCLUST at 98% similarity level. Chimera sequences identified by ChimeraSlayer and singletons were discarded.

Richness and diversity were estimated using rarefaction analysis and the Shannon Diversity Index (H’) based on abundance of OTU sequences. Principal component analysis (PCA) based on Euclidean distance was performed on log 2-transformed relative abundances (normalized for each sample) of OTUs in each sample to visualize whether there is segregation in gut microbiota structure among animals groups. As previously described^32^,^65^, the statistical significance of the segregation among animal groups was assessed by multivariate analysis of variance (MANOVA) test based on PCA scores along principal components of samples in MATLAB R2010a (The MathWorks, Inc., Natick, MA, USA).

To find the OTUs of which abundance was different between two animal groups at individual time points, linear discriminant analysis effect size (LEfSe, http://huttenhower.sph.harvard.edu/galaxy)^66^ was performed with the dataset of log 2-transformed relative abundance of OTUs in each sample. OTUs satisfying the following criteria were picked out: (1) the alpha value for the factorial Kruskal–Wallis test among classes is <0.05 and (2) the threshold on the logarithmic LDA score for discriminative features is >2.0.

Sequences were subjected to the RDP classifier (RDP database version 11.4) for taxonomic assignment at a 50% confidence level.

### Cecal fermentation end products measurement

The concentrations of the short chain fatty acids (SCFAs), including acetate, propionate, butyrate, and n-valerate, and branched chain fatty acids (BCFAs), isobutyrate and isovalerate in the cecal content were determined using an Agilent 6890N gas chromatograph (Agilent Technologies, Wilmington, DE, USA). Two milliliters supernatant was prepared by reconstituting all cecal content of each animal in 0.01 M phosphate buffer solution (PBS) followed by centrifugation at 9000 g for 5 min at 4 °C. The supernatant was acidified with a 1/10 volume of 50% H_2_SO_4_ and extracted with ethyl ether. The concentrations of SCFAs and BCFAs were determined in the organic phase using an Agilent 6890N gas chromatograph (Agilent Technologies, Wilmington, DE, USA) equipped with a polar HP-FFAP capillary column (0.25 mm × 0.25 mm × 30 m) and flame ionization detector (Agilent Technologies, Wilmington, DE, USA). Helium was used as the carrier gas. The initial oven temperature was 140 °C, which was maintained for 10 min and then raised to 165 °C at 5 °C/min, increased to 270 °C at 25 °C/min, and held at this temperature for 2 min. The detector temperature was 280 °C, and the injector temperature was 250 °C. Data handling was performed with an Agilent ChemStation (version G2070AA, Agilent Technologies, Wilmington, DE, USA).

### Statistical analysis of physiological and biochemical data

To test the significance of differences in physiological and biochemical values between animal groups, data with a normal distribution were compared by analysis of variance (ANOVA) test (SPSS Inc., Chicago, IL, USA), and data without a normal distribution were compared by the Mann-Whitney test (MATLAB R2010a). Differences were considered significant when p < 0.05.

## Additional Information

**Accession code:** The sequence information in this paper has been submitted to the GenBank Sequence Read Archive database under accession number SRP064646.

**How to cite this article**: Wang, J. *et al*. The structural alteration of gut microbiota in low-birth-weight mice undergoing accelerated postnatal growth. *Sci. Rep.* **6**, 27780; doi: 10.1038/srep27780 (2016).

## Supplementary Material

Supplementary Information
