# Supplementary material for: The structural alteration of gut microbiota in low-birth-weight mice undergoing accelerated postnatal growth
Source: Sci Rep. 2016 Jun 9;6:27780. doi: 10.1038/srep27780 (PMC4899793; doi:10.1038/srep27780)
Supplement: Supplementary Information [file srep27780-s1.pdf]

The structural alteration of gut microbiota in low birth-weight mice undergoing accelerated postnatal growth

Jingjing Wang, Huang Tang, Xiaoxin Wang, Xu Zhang, Chenhong Zhang, Menghui Zhang, Yufeng Zhao, Liping Zhao, Jian Shen

Supplementary Information

Summary

The supplementary information includes supplementary materials and methods, three supplementary tables and four supplementary figures.

Supplementary Materials and Methods

Measurement of the concentration of serum lipopolysaccharide binding protein (LBP) with Enzyme-linked immunosorbent assay (ELISA)

The amount of lipopolysaccharide binding protein (LBP) in serum was measured with ELISA kits for LBP of mice (Cell Sciences, Canton, MA, USA) according to manufacturer's instructions.

Supplementary Tables

Table S1 Body weight (grams) of offspring mice in three animal groups from birth to age 24 weeks

Age	Control	NB+A	LB+A
**0 day**	1.80 ± 0.02	1.86 ± 0.02*	1.28 ± 0.02***###
**3 day**	2.40 ± 0.10	2.30 ± 0.13	2.36 ± 0.13
**6 day**	4.63 ± 0.22	4.47 ± 0.21	4.39 ± 0.24
**9 day**	6.84 ± 0.22	7.27 ± 0.25	7.23 ± 0.31
**2 week**	9.11 ± 0.17	10.40 ± 0.26**	9.87 ± 0.31*
**18 day**	14.73 ± 0.34	17.79 ± 0.48***	16.72 ± 0.62**
**3 week**	18.00 ± 0.80	22.50 ± 0.56***	21.46 ± 0.77**
**4 week**	28.04 ± 0.42	31.32 ± 0.30***	29.83 ± 0.81*
**8 week**	43.63 ± 0.99	47.25 ± 0.91*	43.42 ± 0.81#
**12 week**	48.17 ± 1.10	52.46 ± 1.21*	47.96 ± 1.25#
**24 week**	53.45 ± 1.47	59.25 ± 1.50*	56.67 ± 1.79

Data are shown as means ± SEM. Differences were assessed by ANOVA. * P < 0.05, ** P < 0.01, *** P < 0.001 *vs.* control; # P < 0.05, ## P < 0.01, ### P < 0.001 *vs.* NB+A.

Table S2 Food intake restriction during gestation reduced maternal weight gain and pups' birth weight.

	Free food access in gestation	50% food intake restriction in gestation
**Dams**		
n	10	12
Food intake after dietary restriction (g/day/mouse)	9.8 ± 0.3	5.2 ± 0.1***
Weight gain on the 17th day of gestation (g)	23.0 ± 1.4	19.2 ± 0.8*
**Pups**		
n	119	127
Number of pups per litter (total)	12 ± 1	11 ± 1
Number of pups per litter (male)	7 ± 1	6 ± 0
Pups' birth weight (g)	1.83 ± 0.01	1.28 ± 0.02***

Data are shown as means ± SEM. Differences were assessed by ANOVA. * $P < 0.05$, *** $P < 0.001$

Table S3 LB+A mice had significantly higher body fat mass and percentage than NB+A and control animals at age 24 weeks

Time	4-week			24-week		
**Offspring Group**	**Control**	**NB+A**	**LB+A**	**Control**	**NB+A**	**LB+A**
**n**	12	12	12	11	12	12
**Body weight, g**	28.04 ± 0.41	31.32 ± 0.30 ***	29.83 ± 0.81 *	51.45 ± 1.34	56.75 ± 1.32 *	53.75 ± 1.75
**Epididymal, g**	0.25 ± 0.01	0.44 ± 0.02 ***	0.40 ± 0.04 ***	1.42 ± 0.16	1.50 ± 0.18	2.43 ± 0.15 ***###
**Perirenal, g**	0.06 ± 0.00	0.10 ± 0.01 ***	0.09 ± 0.01 **	0.59 ± 0.09	0.56 ± 0.08	0.87 ± 0.05 * ##
**Subcutaneous, g**	0.18 ± 0.01	0.24 ± 0.01 *	0.28 ± 0.02 **	0.68 ± 0.06	0.71 ± 0.09	1.01 ± 0.10 * #
**Mesenteric, g**	0.23 ± 0.01	0.25 ± 0.02	0.25 ± 0.02	0.74 ± 0.09	0.87 ± 0.10	1.09 ± 0.09 **
**Epididymal /Body weight, %**	0.88 ± 0.04	1.37 ± 0.05 ***	1.34 ± 0.10 ***	2.72 ± 0.29	2.62 ± 0.30	4.53 ± 0.25 *** ###
**Perirenal/ Body weight, %**	0.20 ± 0.02	0.31 ± 0.02 **	0.31 ± 0.03 ***	1.14 ± 0.16	0.98 ± 0.13	1.63 ± 0.09 * ##
**Subcutaneous/ Body weight, %**	0.66 ± 0.04	0.77 ± 0.04	0.93 ± 0.07 ** #	1.32 ± 0.10	1.22 ± 0.14	1.85 ± 0.13 ** ##
**Mesenteric/ Body weight, %**	0.81 ± 0.03	0.81 ± 0.05	0.82 ± 0.05	1.42 ± 0.13	1.51 ± 0.14	2.01 ± 0.13 ** #

Data are shown as means ± SEM. Differences were assessed by ANOVA. * $P < 0.05$, ** $P < 0.01$, *** $P < 0.001$ *vs.* control; # $P < 0.05$, ## $P < 0.01$, ### $P < 0.001$ *vs.* NB+A.

Supplementary Figures

Figure S1

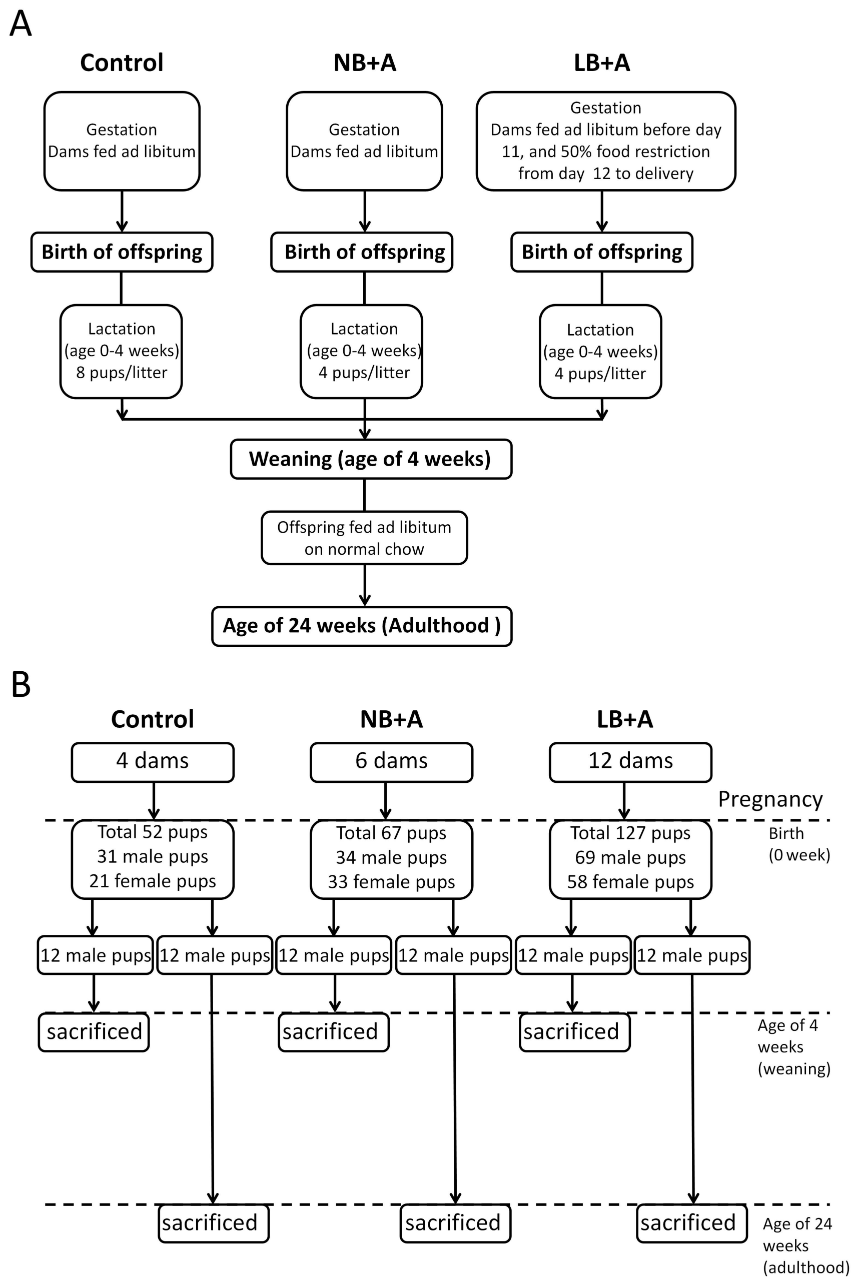

Figure S1 Experimental design of the animal trial. A: Animal grouping. B: The number of dams and offspring in three animal groups.

Figure S2

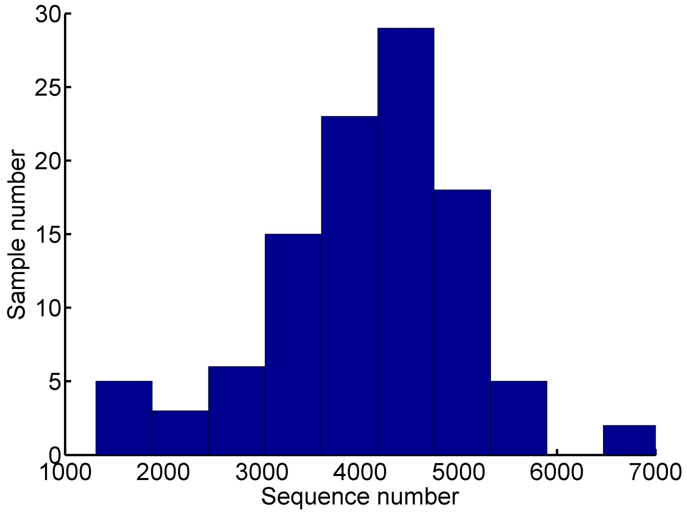

Figure S2 Sample distribution of 432, 428 usable reads in 454 pyrosequencing.

Figure S3

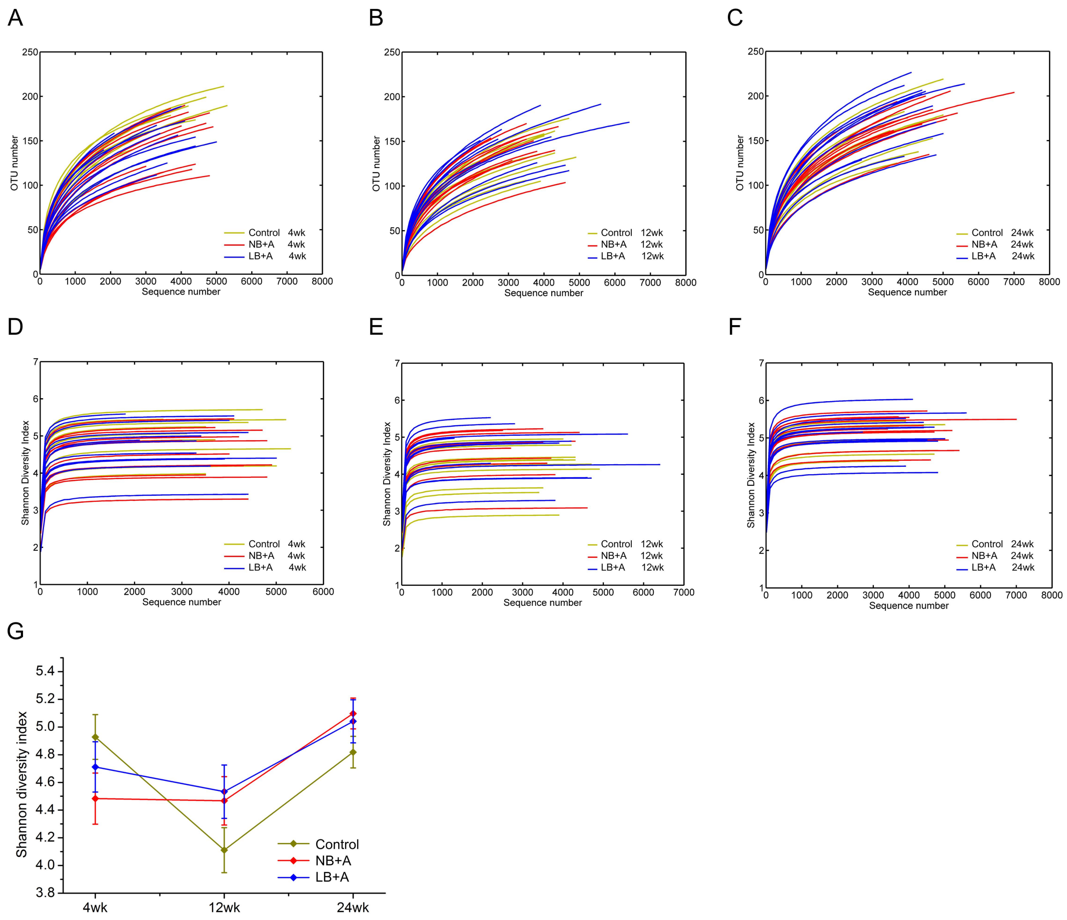

Figure S3 Alpha-diversity analysis of 454 pyrosequencing of fecal samples of three mouse groups at age 4, 12 and 24 weeks. A-C: Rarefaction analysis at age 4 (A), 12 (B) and 24 (C) weeks. D-F: Shannon Diversity Index curves at 4 (D), 12 (E) and 24 (F) weeks. G: Shannon diversity index. Data are shown as means ± SEM. Differences were assessed by ANOVA, but there was no significant difference in Shannon index between any two animal groups at three time points. n=11 for control group at age 4 and 24 weeks; n=12 for control group at age 12 weeks and other two group at three time points.

Figure S4

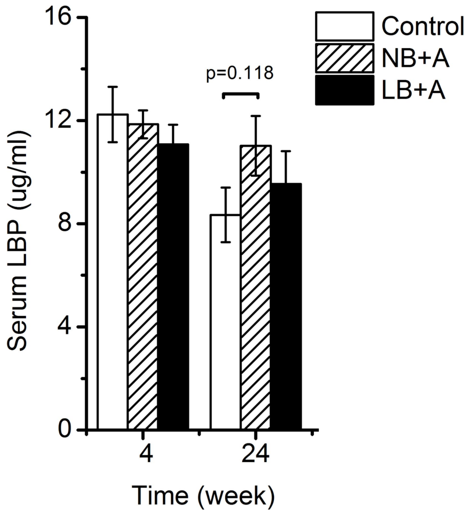

Figure S4 The concentration of serum lipopolysaccharide binding protein (LBP) in three groups of mice at age 4 and 24 weeks. Data are shown as means ± SEM. Differences were assessed by ANOVA. n=11 for control group; n=12 for NB+A and LB+A group.
